# Recurrent stroke syndrome without abnormality on magnetic resonance imaging caused by stenosis of middle cerebral artery: A case report

**DOI:** 10.1016/j.radcr.2022.05.008

**Published:** 2022-06-03

**Authors:** Ajibatul Choriqoh, Achmad Firdaus Sani, Dedy Kurniawan

**Affiliations:** Neurology Department, Universitas Airlangga, Faculty of Medicine Airlangga University, Dr. Soetomo General Hospital, Surabaya, Indonesia

**Keywords:** Hypoperfusion, Middle Cerebral Artery, Stenosis, Stroke, ACA, anterior communicating artery, DSA, Digital subtraction angiography, DWI, Diffusion-weighted imaging, FLAIR, Fluid-attenuated inversion recovery, HDL, high-density lipoproteins, LDL, low-density lipoproteins, MCA, Middle cerebral artery, MRI, Magnetic Resonance Image, PCA, Posterior Communicating Artery, rCBF, regional cerebral blood flow, TTE, transthoracic echocardiogram

## Abstract

Middle cerebral artery stenosis is the leading and the most frequent cause of stroke due to intracranial stenosis in Asia. Magnetic resonance imaging (MRI) is more sensitive than computed tomography of the head for detecting acute brain ischemia. We are reporting a case of a 28-year-old female with recurrent left hemiparesis. After the last attack, an improvement in motor function was seen in less than 24 hours. Though the restoration of motor functions is not complete yet, an MRI scan that was done two weeks later appeared normal. Ischemic stroke in middle cerebral artery stenosis is associated with hemodynamic stroke due to hypoperfusion or lack of blood flow to brain tissue. Recurrent strokes can be prevented by better medical management in patients through regulation and management of risk factors.

## Introduction

Middle cerebral artery (MCA) stenosis is the most crucial vascular lesion causing ischemic stroke, especially in the Asian population. Patients with intracranial stenosis tend to have hemodynamic hypoperfusion, especially under poor collateral circulation [1]. Magnetic Resonance Image (MRI) is increasingly being used as the investigation of choice for diagnosing acute ischemic stroke. Diffusion-weighted imaging (DWI) is sensitive in diagnosing acute cerebral ischemia [2]. However, it is challenging to confirm the stroke diagnosis in a symptomatic patient with a negative DWI. A previous study found that DWI-MRI did not identify relevant ischemic lesions in one-third of patients with nondisabled stroke [3,4]. We report a case of symptomatic MCA stenosis with recurrent stroke in a young woman caused by hypoperfusion or hemodynamic stroke with a normal MRI.

## Case Report

A 28-year-old female patient, with a body mass index of 31, complained of a sudden body weakness on the left side 2 weeks back. She also complained of intermittent moderate-intensity headache on the left side with no nausea or vomiting. She fainted thrice in the last month and also mentioned that three years ago she had body weakness on the left side that resolved spontaneously within 30 min. She was not taking any medication at that time. On examination, there was an improvement in the body weakness on her left side. Though the physical examination was normal, a slight facial palsy of central type was noted on the left side. The motoric scale for left hemiparesis was 4+ for both upper and lower left extremities.

**Fig. 1:**
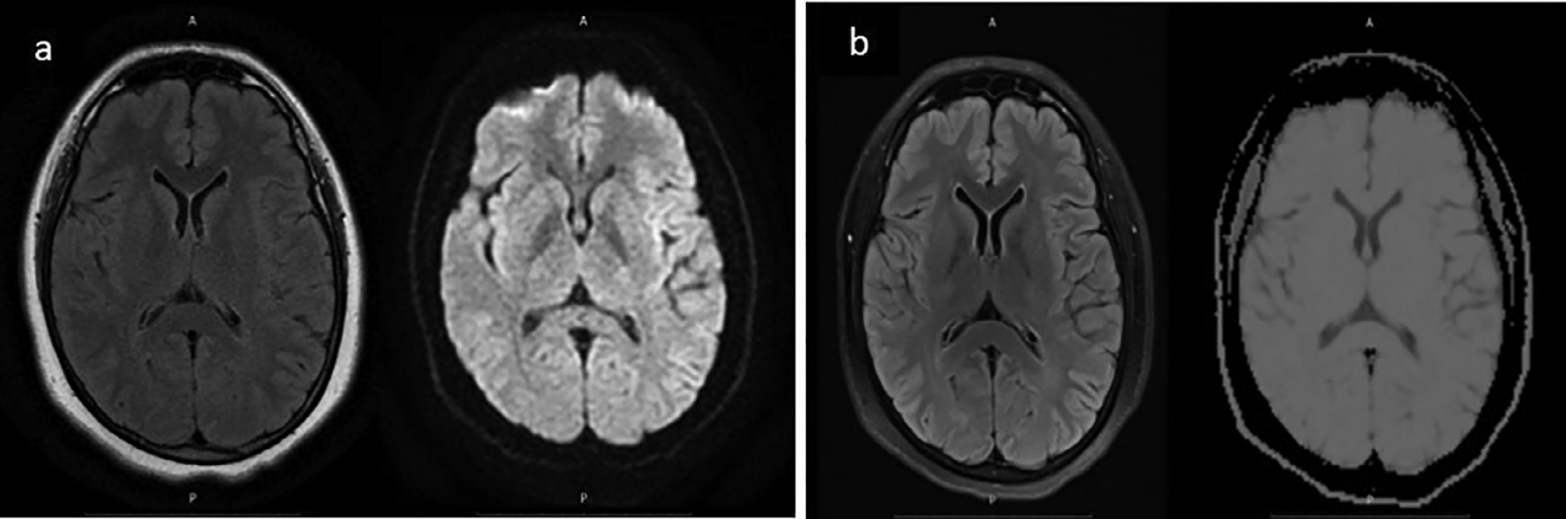
(a) Diffusion-weighted imaging (DWI)/Fluid-attenuated inversion recovery (FLAIR) normal (after the patient's second attack); (b) Normal DWI/FLAIR (after the third attack, 3 months after the first magnetic resonance imaging).

**Fig. 2:**
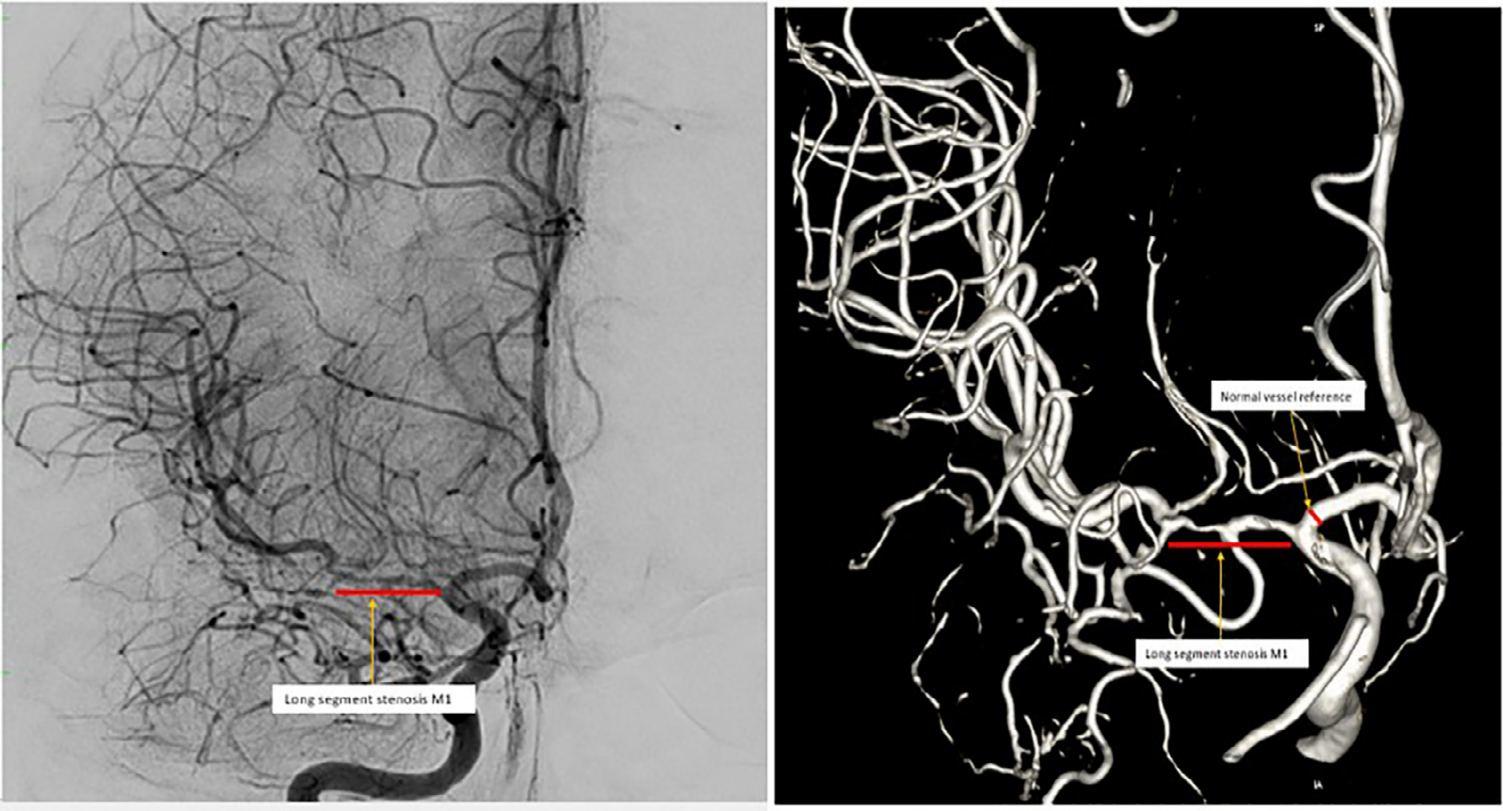
Cerebral digital subtraction angiography images show long segment stenosis in right M1 (2D and 3D images).

Routine laboratory results and homocysteine results were normal. Her lipid profile was, LDL 166 mg/dL (normal range <100 mg/dL), HDL 51 mg/dL (normal range <50 mg/dL), triglyceride 120 mg/dL (normal range <150 mg/dL), total cholesterol 221 mg/dL (normal range < 200 mg/dL). MRI showed no lesions in the brain parenchyma (Fig. 1a). Digital subtraction angiography (DSA) showed 80% stenosis of the right distal M1 with 3 mm of length, with collaterals from the right anterior communicating artery (ACA) and posterior communicating artery (PCA) (Fig. 2). The results of the transthoracic echocardiogram were normal. The patient received dual antiplatelet therapy comprising of acetylsalicylic acid (ASA) 100 mg and clopidogrel 75 mg along with atorvastatin 20 mg.

Two months after receiving dual antiplatelet therapy, she complained of sudden weakness on the left side of her body and slurred speech. These complaints improved within 2 hours spontaneously. At that time, her blood pressure was 110/80 mmHg and the pulse was regular at 84 beats/min. The neurological examination revealed; left central facial palsy, left central lingual palsy, and left hemiparesis with a motoric scale one for the left upper and lower extremities. The motoric scale improved to 4 on the left upper and lower extremities within 24 hours.

**Fig. 3:**
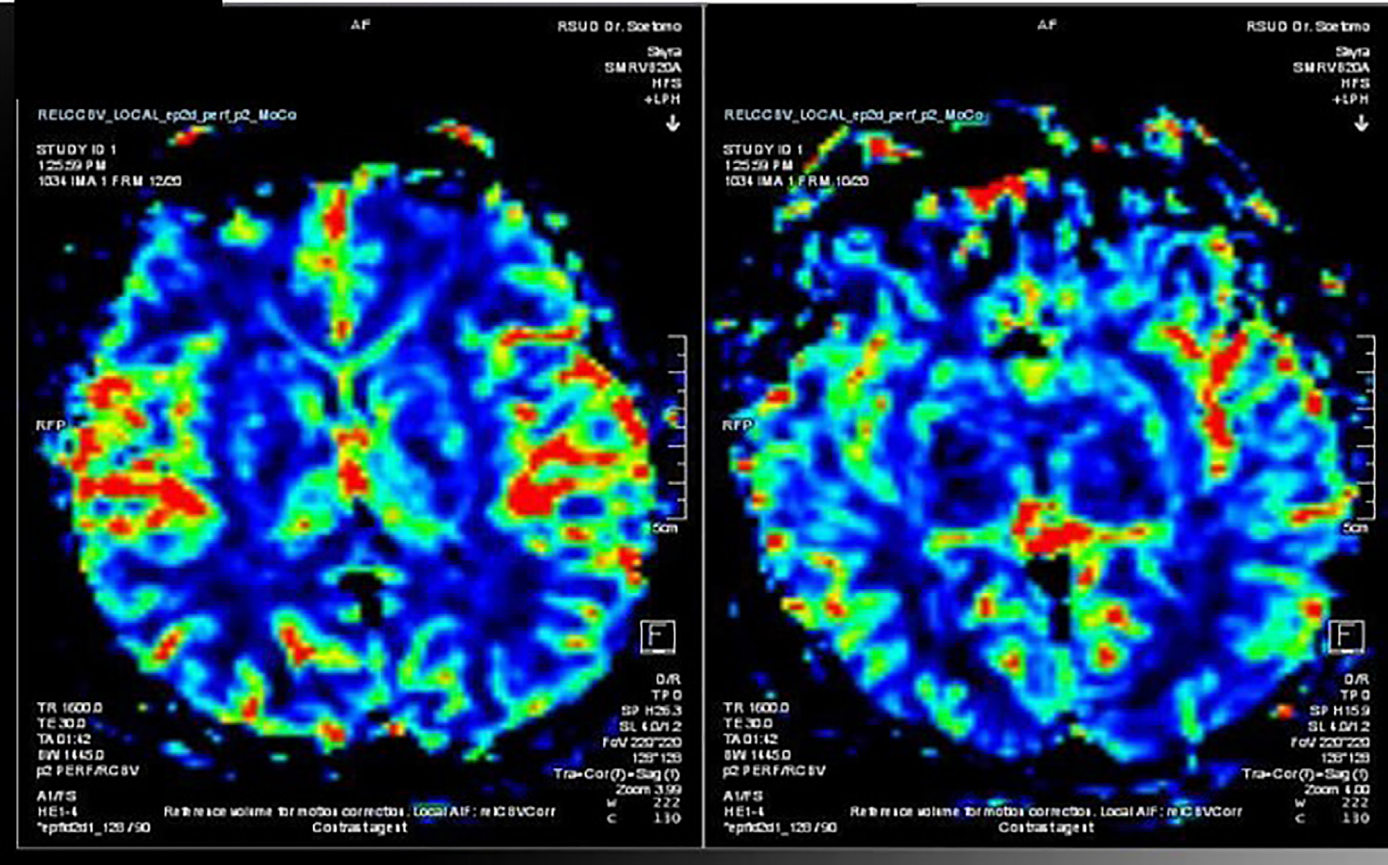
MR perfusion shows lower rCBF as compared to the left side.

Due to recurrent attacks, a magnetic resonance (MR) perfusion scan was done to see the degree of hypoperfusion in the patient. A conventional MRI did not show signs of infarction (Figs. 1b, 3), but an MR perfusion scan showed a decrease in right regional cerebral blood flow (rCBF) as compared to the contralateral side. The management of this patient was to continue dual antiplatelet therapy; however, the dose of atorvastatin was increased to 40 mg.

## Discussion

In this case, the patient is a young woman with right M1 MCA stenosis. The detailed mechanism and natural history of MCA stenosis in young adults remain unclear [5]. However, this patient had hypercholesterolemia, which caused atherosclerotic lesions. A previous study reports that hypercholesterolemia was more common in patients with MCA stenosis than in those without MCA stenosis [6]. Intracranial stenosis is most commonly caused by an atherosclerotic lesion of the intracranial blood vessels, which causes narrowing or blockage of these blood vessels [5].

A neurological deficit in this patient can be a hemodynamic stroke caused by hypoperfusion, which in this case occurred due to a lack of collateral blood supply in the area distal to the occlusion or stenosis of the vessels [1]. Hypoperfusion was proven by the results of MR perfusion, which showed that the right rCBF perfusion was lower than the contralateral side. Stenosis tends to cause vasodilation, which reduces cerebrovascular reactivity and at the same time, reduces the effectiveness of autoregulation. Conventional theory suggests that hemodynamic disturbances result from repeated episodes of hypotension due to severe arterial stenosis or occlusion [7].

The brain MRI picture in this patient was normal after the second episode of stroke and also after three months (Fig. 2). In a study done by Makin et al. [8], it was found that in nearly one-third of patients having a nondisabled stroke, acute ischemic lesions could not be seen on DWI-MRI. DWI is sensitive to acute ischemia [2]. The increased signaling that makes ischemic tissue visible is due to the limited diffusion of water in the extracellular space as water enters from the extracellular space into the intracellular space causing intracellular edema at the onset of ischemia. However, the pathophysiological changes leading to cellular edema suggest that the reduction in cerebral blood flow required to initiate cellular swelling is more severe when focal neurologic deficits are present. Moreover, the likelihood of seeing acute ischemic lesions on DWI-MRI increased with increasing stroke severity and ischemia duration. Therefore, nondisabled strokes might have a decreased blood flow leading to focal neurologic deficits that are not severe enough to cause DWI lesions [4]. Another possible cause of a negative DWI is that the swelling of the cells lasts for a short time, hence an MRI cannot detect it [3,8].

This patient had symptomatic improvement due to good collateral arteries from the right ACA and right PCA, according to DSA results. Acute obstruction induces blood flow through the collateral tissue followed by remodeling and has the potential for the formation of new collaterals [9]. Good collateral circulation also improves the perfusion in the area. Hence, there is a motor improvement in the patient. This might also explain the possibility of having negative DWI lesions like in the study done by Mair et al., which found that DWI lesions became smaller and improved after intravenous thrombolysis [10].

Management of stroke patients with negative DWI lesions should be the same as that of patients with DWI-positive lesions [8]. It is necessary to manage risk factors like hyperlipidemia and obesity in these patients. Dual antiplatelet therapy is essential to prevent recurrent stroke in these patients [8].

## Patient consent

Written informed consent was obtained from the patient for the publication of this case report.

## References

[1] Feng X, Chan KL, Lan L, Abrigo J, Liu J, Fang H (2019). Stroke mechanisms in symptomatic intracranial atherosclerotic disease classification and clinical implications. Stroke.

[2] Brunser AM, Cavada G, Venturelli PM, Olavarría V, Rojo A, Almeida J (2018). Diffusion-weighted imaging determinants for acute ischemic stroke diagnosis in the emergency room. Neuroradiology.

[3] Van de Ven EA, de Kort FAS, Vos JA, Kerklaan JP. (2021). Disabling wake up stroke without lesions on initial diffusion weighted imaging - case report and clinical implications. J Stroke Cerebrovasc Dis [Internet].

[4] Raut T, Patil L, Munshi M, Shrivastava M. (2020). DWI negative large artery acute ischemic stroke : a case report with review of literature. Austin J Cerebrovasc Dis Stroke.

[5] Song X, Xue S, Wu J, Ren Y. (2017). Isolated symptomatic middle cerebral artery stenosis in young adults: clinical prognosis and vascular change. Int J Neurosci.

[6] Jeng JS, Hsieh FI, Yeh HL, Chen WH, Chiu HC, Tang SC (2017). Impact of MCA stenosis on the early outcome in acute ischemic stroke patients. PLoS One.

[7] Cai B, Peng B. (2017). Intracranial artery stenosis: Current status of evaluation and treatment in China. Chronic Dis Transl Med [Internet].

[8] Makin SDJ, Doubal FN, Dennis MS, Wardlaw JM. (2015). Clinically confirmed stroke with negative diffusion-weighted imaging magnetic resonance imaging: longitudinal study of clinical outcomes, stroke recurrence, and systematic review. Stroke.

[9] Alves HCBR, Pacheco FT, Rocha AJ. (2016). Collateral blood vessels in acute ischemic stroke: a physiological window to predict future outcomes. Arq Neuropsiquiatr.

[10] Mair G, Von Kummer R, Morris Z, Von Heijne A, Bradey N, Cala L (2018). Effect of IV alteplase on the ischemic brain lesion at 24-48 hours after ischemic stroke. Neurology.

